# Academic resilience, self-efficacy, and motivation: the role of parenting style

**DOI:** 10.1038/s41598-024-55530-7

**Published:** 2024-03-06

**Authors:** Ye Shengyao, Hashem Salarzadeh Jenatabadi, Ye Mengshi, Chen Minqin, Lin Xuefen, Zaida Mustafa

**Affiliations:** 1 Department of Public Education, Wenzhou Vocational College of Science and Technology, Wenzhou, Zhejiang Province China; 2 Department of Education, Faculty of Social Sciences and Liberal Arts, UCSI University, Kuala Lumpur, Malaysia; 3 Department of Science and Technology Studies, Faculty of Science, Universiti Malaya, Kuala Lumpur, Malaysia

**Keywords:** Social cognitive theory, Academic resilience, Academic motivation, Parenting style, Self-efficacy, Public health, Quality of life

## Abstract

Previous research has found that parenting style influences academic resilience. Nonetheless, few studies have focused on the mechanism underlying the relationship between parenting style and academic resilience. This study aims to examine the relationship between adolescents' parenting style and academic resilience, drawing upon the framework of Social Cognitive Theory. Specifically, it wants to explore the mediating roles of self-efficacy and academic motivation in this relationship. The participants were 518 students chosen at random from educational institutions in the Chinese provinces of Zhejiang, Shanghai, and Jiangsu. Social Cognitive Theory was the theoretical foundation for the study, and the Parental Authority Questionnaire was used to measure parenting style. Out of the respondents, 55.5% were male and 45.5% female. The student allocation in the study sample was as follows: 62.34% undergraduate, 28.22% master’s, and 9.44% PhD. More than 60% of participants were over 25 years old. Moreover, the findings revealed that parenting style was directly and positively related to academic resilience. Parenting style was also found to be indirectly and positively related to academic resilience via self-efficacy and academic motivation, respectively, and sequentially. More crucially, it was discovered that the direct association was far lower than the indirect effects, with self-efficacy being the most effective. The study indicates a relationship between parenting style and academic resilience in adolescents, with self-efficacy and academic motivation acting as the main mediators. These findings emphasize the significance of these intermediary elements, implying that they play a larger role than the direct influence of parenting style alone.

## Introduction

People who effectively continue their academic lives despite challenges in life are examples of resilient people. According to Greene^1^, the concept of resilience is derived from the Latin term “resiliens”, which indicates the flexibility of matter and the possibility of easily returning to its former shape. The concept of resilience can be understood in a number of different ways depending on the social setting. In the field of psychology, for example, it is described as the approaches that individuals choose in order to react to a difficult situation^2^. On the other hand, in the field of education, it is concerned with the students' capacity to deal with challenges and be successful^3^. In a broader sense, the phrase refers to an efficient strategy that can be utilised to triumph over challenging circumstances^3^. Academic resilience refers to the ability of individuals to achieve academic success and demonstrate high-level performance, especially in the face of challenging life circumstances and adverse living conditions that may otherwise predispose them to academic failure and dropout^4^. Academic resilience, as defined by Brewer, Van Kessel^5^, is the likelihood of achieving academic success in spite of adverse experiences, unfavourable environmental situations, and the absence of initial personality qualities.

### Self-efficacy

Self-efficacy, a core notion in Albert Bandura's social cognitive theory, is essential in psychology and human behavior^6^. It refers to an individual's belief in their ability to execute the actions required to achieve specified performance goals. Essentially, it is a person's confidence in their capacity to succeed in a certain scenario or complete a task. This confidence in one's own competence influences many areas of human behavior, including as the decisions we make, the effort we put into activities, how long we endure in the face of adversity, and how we feel about our accomplishments and failures^7^. High self-efficacy can lead to a more resilient and resolute response to problems, whereas low self-efficacy might result in a lack of confidence and a proclivity to give up easily^8^. Self-efficacy influences not just our behaviours, but also our cognitive processes, resulting in either a positive or negative feedback loop in our behavior.

Self-efficacy in the academic setting is crucial, especially among university students, because it has a substantial impact on their attitude to learning, academic achievement, and overall educational experience. University students with strong academic self-efficacy set higher objectives and are more devoted to obtaining them because they believe they can succeed in academic assignments^8^. This conviction in their own academic talents allows students to dive deeper into their studies, use effective learning tactics, and persevere in the face of obstacles and setbacks. High self-efficacy is also associated with improved stress management and coping skills, as students feel empowered and less overwhelmed by academic obligations^9^. Furthermore, it promotes a positive attitude toward learning and problem solving, which is vital in a university setting where autonomous study and critical thinking are valued^10^. This self-confidence in their academic abilities can lead to increased academic accomplishment and satisfaction, influencing their future job pathways and life chances. Nurturing academic self-efficacy is vital for university educators and counsellors since it not only improves students' academic achievement but also contributes to their whole personal and professional development.

## Academic motivation

Motivation is an important factor in human behavior, acting as the driving force behind human behaviours, decisions, and objectives. It is a complex interaction of biological, emotional, social, and cognitive variables that drives us to behave in specific ways. Depending on where the incentive comes from, it might be intrinsic or extrinsic^11^. Intrinsic motivation comes from within the individual. It is motivated by personal gratification, interest, or enjoyment in the activity itself. Extrinsic motivation, on the other hand, stems from outside influences or rewards. This could be money, recognition, grades, or admiration^11^.

Academic motivation is extremely important, especially among university students. University is an important period in which students prepare for their future careers and personal growth. Academic motivation is critical during this era because it directly affects students' learning effectiveness, academic achievement, and ability to deal with academic stress and problems^12^. Highly motivated students are more likely to engage thoroughly in their studies, contribute to class, and strive for academic distinction. They are also better prepared to deal with the rigors of university life, such as the often-difficult academics and the requirement for excellent time management. Furthermore, strong academic drive is tightly related to career goals, allowing students to overcome obstacles and setbacks^10^.

### Role of parenting style

The parenting style, being the primary determinant of the type of home environment that a kid is exposed to while growing up, plays a vital part in the development of resiliency in a student. Parenting style is a significant challenge for study scholars. Several theories exist on parenting style*. Baumrind's Parenting Styles* theory, founded by Baumrind^13^, is one of the earliest and well-organized theories. This theory distinguishes three major parenting styles based on responsiveness and demandingness: authoritative, authoritarian, and permissive. *Attachment Theory*; is developed Bowlby^14^. In this theory the importance of an emotional relationship between a child and their primary caregivers is highlighted, as is the impact on emotional and social development. *Social Learning Theory of Parenting*, developed by Bandura and Walters^15^. This theory emphasizes the importance of observational learning, imitation, and modelling in the development of child behavior, as well as the influence of parents as role models. *Parental Acceptance-Rejection Theory*, developed by Rohner^16^. This hypothesis focuses on the impact of parental approval or rejection on children's development, arguing that perceived rejection can lead to psychological maladjustment. When the many parenting style theories are compared, distinct viewpoints and emphases emerge for understanding the complex dynamics of child-rearing and its impact on child development.

Parenting style theories stress that the success of various parenting styles can vary dramatically across different phases of a child's development, emphasizing the significance of adjusting parenting strategies as children grow. Attachment Theory^14^, for example, emphasizes the importance of a stable emotional link in infancy, as well as responsive and attentive caring. Specific parenting style ideas are especially relevant for university students as they transition to maturity and increasing independence^17^. At this point, Baumrind's authoritative parenting principles^13^ remain important; offering emotional support and guidance while respecting the young adult's increasing autonomy is critical. Parenting university students is a delicate combination of assistance and independence, allowing them to develop self-reliance and follow their own path while knowing they have a solid support system to turn to^18^. Specific parenting style ideas are quite useful for university students, especially when it comes to promoting academic resilience. Baumrind's authoritative style is extremely relevant; it mixes support and encouragement of independence, which is critical for pupils confronting academic obstacles^19^. This technique helps to develop self-efficacy and problem-solving skills, which are necessary for resilience in a difficult academic setting. For example, Mullins, Zhou^20^ found that authoritative parents likely to have a beneficial correlation with their children's academic resilience^21^. They set clear expectations and provide a nurturing and encouraging environment. These parents provide structure, encouragement, and emotional support. They teach in their children the self-discipline, time management, and problem-solving techniques that are crucial for academic success. Parents who are in charge typically give their children the confidence to handle obstacles in school^22^. Authoritarian parents frequently have high academic standards, but they might not give their kids the emotional support and autonomy they need to grow up resilient^23^. Such circumstances can make it difficult for students to be adaptable and deal with disappointments. On the other hand, parents who are encouraging frequently have a favourable correlates with academic resilience. They offer a supportive and nurturing environment. These parents provide their kids with emotional help and support, which can increase their motivation and self-esteem^24^. When they have a solid support structure, students feel more equipped to handle academic obstacles. It became obvious that parenting styles that balance support and expectations tend to help college students develop more academic resilience. However, academic resilience is also greatly influenced by the unique qualities of each student and the larger university environment^25^.

Research has indicated that the type of parenting a child receives has a correlation with their ability to persevere academically, an area in which self-efficacy is a potential predictor^26,27^. Self-efficacy can be defined as "an individual belief in one's capabilities to organise and execute the courses of action required in producing given attainments"^28^. Self-efficacy can be developed through a variety of methods. According to another definition, it is "the perception of one's ability to successfully perform a particular behaviour"^29^. According to the findings of certain research by Romano, Angelini^30^, academic desire is also a possible predictor of academic resilience. Academic motivation can be defined as the human capacity to overcome acute or chronic hardship in resilience^31^, or the ability to effectively deal with academic setbacks, obstacles, adversity, and pressure. However, almost no research has empirically investigated the ways in which the parenting style of adolescents is connected to the academic resilience of students by examining the roles of self-efficacy and academic motivation as mediators.

### Parenting style and academic resilience

Relevant research has revealed that parenting style has a correlation with academic resilience^32^. The parenting styles of the students' parents as well as the connections made throughout infancy and adolescence may still have a correlation with their academic resilience even after they have attained a certain amount of independence and autonomy at the university level. Researchers Aliyev, Akbaş^33^ found that different parenting methods can have a correlation with child's academic resilience, which is defined as the capacity of a student to triumph over obstacles and failures encountered while pursuing academic goals. Several pieces of research have pointed to the parenting style as one of the primary factors that determines a student's ability to persevere academically^34,35^.

According to Romano, Angelini^30^, university students with supportive parents have excellent emotional and academic support systems, which can contribute to their academic resilience. Similarly, Rahiem^36^ found that supportive parents can enhance their students' confidence and motivation, allowing them to tackle academic challenges more efficiently. On the other hand, university students who have helicopter parents may struggle to develop autonomy and problem-solving skills^37^. It has been suggested that because they are accustomed to parental assistance, they may be less resilient when faced with adversity. These students may have difficulty making decisions and accepting responsibility for their academic progress. It is critical to understand that university students are increasingly responsible for their academic and personal lives, and the influence of parenting style may reduce as they develop independence. The following hypothesis is suggested based on this point of view.

#### H1

Parenting style is positively associated with students’ academic resilience.

### Self-efficacy and academic motivation

The relationship between self-efficacy and academic motivation has proven challenging to model in educational psychology. There are four categories of literature on the relationship between self-efficacy and academic motivation. Some of the studies examined the linkage between self-efficacy and academic motivation in this direction: self-efficacy → academic motivation^10,38,39^. Students with high self-efficacy are more likely to establish ambitious academic goals and demonstrate a strong commitment to reaching them. This confidence in their talents inspires people to participate more thoroughly in learning activities, employ effective learning tactics, and persevere in the face of adversity. Second group beliefs differ are also certain beliefs that differ from those of the first category. In their framework, they determined the following relationship “academic motivation → self-efficacy”^40,41^. In this school of thought, maybe students that are highly motivated are more likely to engage in academic activities with excitement and tenacity. This greater engagement frequently results in improved performance and favourable outcomes, which reinforces a student's confidence in their talents. Successful experiences, motivated by high motivation, are important factors in developing self-efficacy. The third group only considers the relationship, without considering cause and effect, between self-efficacy and academic motivation^8^. In the last group of studies, which are few, they concluded both self-efficacy and academic motivation for estimating dependent variables; however, they didn’t show correlations in their framework^42^.

Understanding the relationship between self-efficacy and academic motivation is critical to comprehending student learning and achievement. The relationship between self-efficacy and academic motivation may be more important than simply examining the influence of self-efficacy on academic drive. This is because the correlation takes a larger view, revealing how these two characteristics interact and influence each other in academic settings. Therefore, this study intends to test the following hypothesis:

#### H2

There is a significant correlation between Self-efficacy and academic resilience.

### Self-efficacy as a mediator

In social cognitive theory, Bandura^6^ has placed emphasis on how self-efficacy is built and how resilience is affected by it. Self-efficacy, often known as an individual's belief in their own capacity to succeed in specific academic activities, plays a crucial waves in the ability to anticipate academic resilience^43^. According to Huang, Ding^44^, students who have a high level of self-efficacy are more likely to be academically resilient, since they are more likely to approach academic challenges and failures with confidence. They have a stronger belief that they are able to overcome and over adversity as a result of having the talents and capacities necessary to successfully accomplish so. Wu, Fan^45^ found that high levels of self-efficacy are linked to increased levels of motivation and persistence in academic achievements. When students have confidence in their talents, they are more likely to set lofty goals for themselves, work hard to accomplish those goals, and remain resolute in the face of challenges. The ability to remain focused and persistent in one's studies are essential components of academic resilience. Uygur, Asici^32^ came to a similar conclusion and pointed out that the parenting style of an adolescent is a significant factor in their sense of self-efficacy, which in turn influences their academic resilience. According to the perspectives presented above, self-efficacy may indirectly influence parenting style, which could be associated with adolescents' academic resilience. The following hypotheses are presented as a result of these considerations:

#### H3

Parenting style is positively associated with academic self-efficacy.

#### H4

Self-efficacy plays a mediating role in the association between parenting style and academic resilience.

### Academic motivation as a mediator

The way in which parents interact with their children might inspire academic motivation. It has been posited that parenting style may play a role in shaping academic motivation within the context of the educational journey^46^. Researchers Jan, Salik^47^ and Lee and Datu^48^ highlighted the fact that the parenting style of the child was a reliable predictor of academic desire. University students who had parents who were authoritative figures in their lives typically maintain high levels of academic drive throughout their time in university^49^. Due to the fact that these characteristics were encouraged throughout their development, it is possible for them to demonstrate self-discipline, a strong work ethic, and a belief in their own skills. Students who fit this profile are more likely to be intrinsically motivated, to have self-defined academic goals, and to be interested in personal development. Lan and Wang^50^ have also proposed that students' academic motivation may be inspired by relational resources, particularly with their parents. As a result, these scraps of evidence support the hypothesis that the parenting style of an adolescent's parents can boost that student's academic motivation.

Academic motivation has a role in the academic resilience of university students. According to Nugraha, Fachrian^51^, students who are academically motivated have a tendency to demonstrate higher levels of accomplishment despite the risks and difficulties involved. Mohan and Verma^52^ and Ayasrah and Albalawi^53^ have demonstrated that one's academic motivation, and more especially their goal orientation, can have a link with their level of resilience. Students who have a focus on mastering are more likely to be able to bounce back from academic setbacks. They place more of an emphasis on growth and learning than they do on the results of performance. Students who have this mindset are more inclined to see challenges not as failures but as chances for personal development. According to Yan and Gai^54^, students who have a higher level of academic motivation also have a higher level of academic resilience. Bülow, Neubauer^55^ provided evidence that kids who report feeling a stronger connection with their parents’ parenting approaches have greater levels of academic motivation. Academic motivation is an essential component of academic resilience. This study therefore hypothesises that there is a positive relationship between academic motivation and academic resilience among adolescents, and that academic motivation may play a mediating role between parenting style and academic resilience.

Self-efficacy can influence students' ability to self-regulate their learning. Students with high level of self-efficacy are more likely to be motivated to plan, monitor, and change their learning strategies. Self-efficacy is a significant predictor of academic motivation^56–58^, which provides a foundation for the serial variables of self-efficacy and academic motivation. The interaction of parents, according to social cognitive theory, enhances the self-efficacy of adolescents. Students who have higher levels of self-efficacy are better equipped to confront challenges, engage themselves in tough learning tasks, and cultivate their academic motivation^27^. Moreover, academic resilience is affected by personal characteristics such as self-efficacy and academic motivation^8^. Accordingly, the connection between parenting style and academic resilience is thought to be able to influence academic resilience via the sequential variables of self-efficacy and academic motivation.

Following the research presented above, the purpose of this study is to investigate the possibility that some aspects of a parent's parenting style may, in fact, favourably influence academic resilience through the roles played by sequential self-efficacy and academic motivation as mediators. Given this, the following possibilities are put forward for consideration:

#### H5

Parenting style is positively associated with academic motivation.

#### H6

Self-efficacy is positively associated with academic motivation.

#### H7

Academic motivation is positively associated with academic resilience.

#### H8

Academic motivation plays a mediating role in the association between parenting style and academic resilience.

### Chain mediating of self-efficacy and academic motivation

Self-efficacy and academic resilience are two psychological notions that are frequently examined in terms of education and personal development. While they are independent concepts, there is a possible link between them because they both contribute to an individual's capacity to navigate and flourish in academic contexts.

Higher levels of self-efficacy are typically related with improved academic achievement and persistence. Students who believe in their abilities to succeed are more likely to set ambitious goals, put in effort, and endure in the face of adversity.

#### H9

Self-efficacy and academic motivation play a chain mediating role in the association between parenting style and academic resilience.

**Figure 1:**
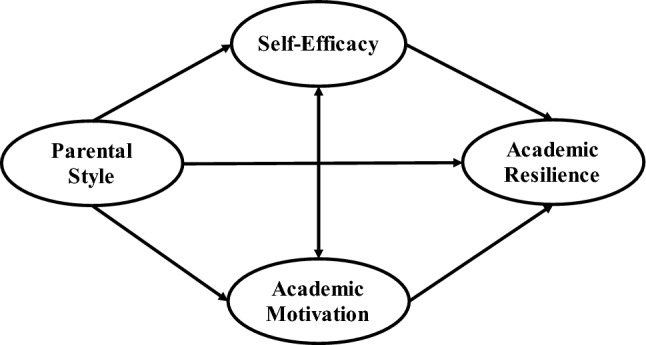
Conceptual framework.

Supported by Social Cognitive Theory and the aforementioned nine hypotheses, a theoretical framework has been developed to examine the correlation between parenting style and academic resilience (See Fig. 1).

## Current study

Without a doubt, parenting style principles are critical to adolescents' academic resilience. In educational research, the association of self-efficacy with academic motivation (self-efficacy → academic motivation) and vice versa (academic motivation → self-efficacy) has been studied. Furthermore, other studies investigated the correlation between parenting style and academic resilience via self-efficacy and motivation individually. They have, however, been examined in parallel, and their possible links have not yet been investigated in a single study. As a result of the underlying gaps, the researchers of this study created a model of their probable relationships.

In light of this, the current research aims to investigate the following:

1. Parenting style has a significant association with both self-efficacy and motivation.
2. There is an interconnection (correlation) between self-efficacy and motivation.
3. Both self-efficacy and motivation have a significant association with academic resilience.
4. Self-efficacy and academic motivation are a chain mediator between parenting style and academic resilience.

The following are some of the contributions that the study makes. First, the study applies Social Cognitive Theory to the Chinese environment in order to investigate the association between parenting style and academic resilience. The results of this investigation provide evidence for the research that has been done on related topics in other countries. Second, the research investigates the mechanism that exists between the types of parenting styles and academic resiliency. This is done by placing an emphasis on the chain-mediating functions that self-efficacy and academic motivation play. According to are new viewpoint, the academic resiliency of students at Chinese universities is primarily determined by personal characteristics like as self-efficacy and academic motivation, which are in turn influenced by environmental factors such as the parenting style of the students' parents.

## Methodology

### Social cognitive theory

Bandura^28^ is credited with developing a theory called Social Cognitive Theory. This theory is a comprehensive framework that places an emphasis on the interaction of personal variables, environmental influences, and behaviour in influencing human development and learning ^28^. According to this school of thought, individuals don't just learn from their own experiences but also from witnessing and modelling the behaviours, attitudes, and results that occur in the lives of others. According to Bandura^59^, human behaviours are both motivated and regulated by a combination of environmental, personal, and behavioural factors. This means that human behaviours are influenced by a combination of all three. Both the social support and the barriers that the environment provides are examples of environmental factors. According to Dubovi and Sheu^60^, personal characteristics such as knowledge, self-efficacy, and outcome expectations are connected with the adoption of behavioural change. According to Bandura^59^, one of the most important personal determinants is a person's belief in their own ability to bring about the desired behavioural changes. Bandura^59^ is credited with the development of the idea of self-efficacy, which describes an individual's belief in their own capacity to effectively complete a given job or accomplish a certain objective. A high sense of self-efficacy is linked to increased levels of both motivation and tenacity in the pursuit of one's goals. According to Wang, Lee^12^, behavioural elements include making an effort or making plans in order to carry out a behaviour. A number of researchers have used social cognitive theory to investigate academic resilience among college students^61–63^. However, not a lot of research has been done to investigate the interconnected relationships between the social cognitive theory and the elements that influence students' academic resilience.

### Measures

The present study employed measurement scales that have undergone prior testing and validation by other researchers. The students' replies were assessed via a 5-point Likert scale that spanned from 1 (indicating strong disagreement) to 5 (indicating strong agreement). Table 1 provides a comprehensive summary of the theoretical literature and research inquiries related to latent variables.

**Table 1:** Literature on how to measure latent factors and the number of questions.

Latent variable	Quantity of inquiries	Theoretical support
Parenting style	30 items	Buri^64^
Self-efficacy	10 items	Rowbotham and Schmitz^66^
Academic motivation	33 items	Harter^65^
Academic resilience	30 items	Cassidy^67^

There are two methods for assessing latent variables: (a) Dimension Scores, and (b) Random Parcelling. This method, dimension method, creates latent variables based on observable data that are thought to represent underlying dimensions. Random parcelling is the process by which numerous observed variables are randomly grouped (or "parcelled") to produce latent variable indicators. The notion here is that merging numerous things into a parcel reduces measurement error while increasing the reliability of the latent construct. We used dimension scores since each latent variable was defined in prior investigations.

Parenting style: To assess parenting styles, psychologists and developmental researchers employ a variety of well-known questionnaires. Diana Baumrind, a developmental psychologist, developed the well-known parenting styles paradigm in the 1960s^13^. She identified three main parenting styles based on two dimensions: parenting responsiveness (or warmth) and parental demandingness (or control). The Parental Authority Questionnaire (PAQ), created by Buri^64^, is based on Baumrind's parenting styles (authoritative, authoritarian, and permissive) and is one of the most extensively used measures for assessing perceived parental authority. The scale has 30 items, each evaluated on a Likert scale from 1 (strongly disagree) to 5 (strongly agree). The measure was slightly modified to be appropriate to people from either single- or two-parent household. The original measure includes distinct metrics for both dads and moms. Participants in the current study chose which parent they wanted to complete the measure with. The PAQ scores range from 10 to 50, with higher scores suggesting a stronger level of the parenting style prototype being examined. The current study's reliability coefficients indicate strong reliability for the three PAQ subscales: authoritarian (α = 0.87), authoritative (α = 0.81), and permissive (α = 0.76), which are consistent with the original measure’s range of α = 0.74 to α = 0.87^64^.

The factor loading for all survey questions ranged between 0.623 and 0.867. The construct reliability (CR) value of the scale was 0.823, which exceeded the evaluation threshold of 0.60. The scale's average variance extracted (AVE) value was 0.581, which exceeded the evaluation requirement of 0.5. This suggests that the scale has a high degree of construct validity and discrimination. The scale's goodness of fit test yielded the following results: GFI = 0.901, AGFI = 0.922, NFI = 0.936, IFI = 0.914, CFI = 0.955, and RMSEA = 0.041 indicate a satisfactory goodness of fit for the scale.

Academic Motivation: Harter^65^ developed a scale with 33 elements to assess academic motivation. Academic motivation is measured using two subscales: intrinsic and extrinsic motivation. Intrinsic motivation refers to engaging in an activity for the sake of intrinsic satisfaction and personal reward. It indicates that students are motivated to learn, explore, and acquire new abilities in an academic setting. This 17-item motivation scale consists of (a) curiosity (3 items), (b) challenge (9 items), and (c) autonomous mastery (5 items). Extrinsic motivation entails engaging in an activity in order to obtain a distinct consequence, such as incentives, grades, or approval from others. This 17-item motivation scale covers (a) pleasing the teacher (4 items), (b) dependence on the teacher (6 items), and (c) easy work (6 items). This scale's minimum and highest scores are 33 and 165, respectively. Values ranging from 33 to 66 indicate a low level of academic motivation, values 66–99 show an average level of academic motivation, and values greater than 99 indicate a strong level of academic drive.

Every survey question had a factor loading that varied from 0.654 to 0.891. The scale's construct reliability (CR) rating was 0.793, above the evaluation cutoff point of 0.60. The AVE value of the scale was 0.659, over the 0.5 evaluation threshold. This implies that the discrimination and construct validity of the scale are quite strong. The following outcomes of the scale's goodness of fit test were obtained: For the scale, the following values of GFI, AGFI, NFI, IFI, CFI, and RMSEA suggest a sufficient goodness of fit: 0.934, 0.951, 0.898, 0.927, and 0.036.

Self-efficacy: Self-efficacy is determined by Rowbotham and Schmitz^66^ based on 10 items. Here are some examples of questions (1) “*I know that I can finish the assigned projects and earn the grade I want, even when others think I can’t*”, (2) “*I know that I can maintain a positive attitude toward this course even when tensions arise*” and (3) “*I am confident in my ability to learn, even if I am having a bad day.*”

Each survey question for this construct—self-efficacy—had a factor loading ranging from 0.711 to 0.839. Construct reliability (CR) of the scale was rated at 0.811, higher than the evaluation cutoff limit of 0.60. The scale's AVE score was 0.681, which is higher than the 0.5 evaluation criterion. This suggests that the scale has very good discrimination and construct validity. The goodness of fit test results for the scale were as follows: A adequate goodness of fit is suggested for the scale by the following GFI, AGFI, NFI, IFI, CFI, and RMSEA values: 0.909, 0.955, 0.931, 0.919, and 0.048.

Academic resilience: Cassidy^67^ proposed a 30-item motivation assessment organized into three unique subscales that includes: 1) Negative affect and emotional response (7 items), 2) Reflecting and adaptive help-seeking (9 items), and 3) Perseverance (14 items).

Factor loadings for the survey questions related to this construct (academic resilience) ranged from 0.639 to 0.863. The scale's construct reliability (CR), which is higher than the evaluation cutoff limit of 0.60, was scored at 0.778. The AVE score of the scale was 0.701, over the assessment requirement of 0.5. This implies that the construct validity and discrimination of the scale are excellent. The scale’s goodness of fit test findings was as follows: The following GFI, AGFI, NFI, IFI, CFI, and RMSEA values indicate that the scale has a satisfactory goodness of fit: 0.944, 0.965, 0.894, 0.976, and 0.023.

### Preliminary study and sampling design

A preliminary investigation was undertaken by distributing a total of 100 questionnaires to students majoring in business at various educational institutions located in the provinces of Zhejiang, Shanghai, and Jiangsu, China. A total of 83 valid responses were obtained, resulting in a response rate of 83%. The preliminary findings from the pilot survey suggest that the measuring constructs shown satisfactory levels of reliability and validity. A power analysis was conducted using the G*Power software, which determined that a minimum sample size of 512 individuals is necessary for the investigation. This calculation was based on an anticipated effect size of 0.2, an assumed alpha value of 0.05, and an estimated power of 0.85. The surveys were initially composed in the English language, and the translation underwent a thorough review by two individuals who are proficient in both Chinese and English. This process involved employing a translation and back-translation technique to ensure accuracy. Additionally, a total of 550 paper-and-pencil surveys were distributed to participants, out of which 528 were successfully retrieved, resulting in a response rate of 96%. Ten items were excluded from the analysis due to incomplete information, resulting in a final sample size of 518 responses.

Out of the responses obtained, 55.5% were classified as male, while 45.5% were classified as female. The allocation of students within the study sample was as follows: The distribution of students in the sample was as follows: 62.34% were classified as undergraduate students, 28.22% were categorised as master's students, and 9.44% were identified as PhD students. The age categories that were observed in the study were classified in the following manner: The age distribution of the participants was as follows: 18–25 years old accounted for 33.26% of the sample, 26–35 years old represented 36.75% of the participants, 36–45 years old comprised 23.67% of the respondents, and persons aged 45 and above constituted 6.32% of the population. Regarding the allocation of academic disciplines, the School of Public Administration constituted 24.56%, the School of Management constituted 23.76%, the School of Economics constituted 22.35%, and the School of Finance constituted 29.33%. Histograms of demographic variables are presented in Supplementary Figs. S1, S2, and S3.

### Ethical approval and consent to participate

The survey was conducted with the University of Malaya Research Ethics Committee approval. The research methods were performed in accordance with the relevant guidelines and regulations. Participants of the study were informed about the purpose, objectives, and their right to participate, decline participation, or withdraw their participation in the research activities by verbal. Respondents have been notified that the information given was private and confidential which only going to use for academic purposes only. Written informed consent was obtained from all respondents.

## Results

Path analysis and mediation analysis were the key statistical tools that were used in this investigation. Path analysis in Structural Equation Modelling (SEM) is a statistical technique that enables researchers to investigate complex interactions among variables. Essentially, it extends simple regression models to allow for the simultaneous examination of many relationships, including direct and indirect effects. In path analysis, route analysis entails drawing a figure (path diagram) that visually depicts these correlations, with arrows indicating the direction of linkage between variables. This method is particularly effective for assessing theoretical models that propose precise patterns of correlations, because it can validate or contradict ideas about how variables are interrelated. Mediation analysis is used to understand how an independent variable can influence a dependent variable. In a nutshell, researchers benefit from doing mediation analysis because it enables them to explore the process or pathway by which direction comes about.

According to Fornell and Larcker^68^, there are several conditions that need to be satisfied in SEM analysis to assess the survey's validity and reliability. In order to be deemed valid, each latent variable within the study must possess a Cronbach's alpha coefficient of 0.7 or above. Based on the findings presented in Table 2, it can be observed that the Cronbach's alpha values for each latent variable meet the established criteria, hence providing support for the validity of this study. Moreover, Average Variance Extracted (AVE) is a widely recognised measure of reliability. Segars^69^ recommended that in order to achieve approval in terms of dependability, the value of this index should exceed 0.5. This indicator successfully aligns with the desired principles and standards. Consequently, the reliability of the study model is validated. Prior to assessing the structural model, it is imperative to ascertain the absence of any linear connection between the constituent pieces. Hair, Black^70^ considered Variation Inflation Factor (VIF) values below 5 as acceptable.

**Table 2:** Analysis of reliability, validity, and multicollinearity.

Variaibles	Cronbach Alpha	AVE	VIF
Parenting style	0.811	0.588	[3.21, 4.11]
Self-efficacy	0.756	0.654	[3.45, 4.32]
Academic motivation	0.802	0.709	[3.09, 4.77]
Academic resilience	0.798	0.767	[3.66, 4.39]

### Fitting model

Fit values above 0.9 indicate a good research model. This study's GFI, RFI, IFI, CFI, TLI, and NFI scores are acceptable. Moreover, the value of RMSEA is less than 0.05. Therefore, this research's model-data fit is accepted (see Table 3). Moreover, TLI has the highest among fitting indices. If the TLI is the highest of the fit indices assessed, it indicates that the proposed model fits the data better than a null model (often a model with total independence among variables). This is because TLI penalizes model complexity, therefore a high TLI shows that any increased complexity in the model is justified by a proportional increase in model fit.

**Table 3:** Fitting indices.

Index	Value
GFI	0.916
RFI	0.927
IFI	0.919
CFI	0.919
TLI	0.936
NFI	0.909
RMSEA	0.046

### Structural model

According to the findings presented in Table 4, the hypotheses H1, H2, H3, H5, H6, and H7 exhibited statistical significance, indicating that their corresponding routes were substantiated by the empirical data. The results of the study indicate that there is a significant and positive relationship between parenting style and academic resilience (β = 0.396, *p* < 0.001), confirming Hypothesis 1. Additionally, academic self-efficacy was found to be significantly and positively associated with academic resilience (β = 0.531, *p* < 0.001), verifying Hypothesis 2. Furthermore, a significant and positive relationship was found between parenting style and academic self-efficacy (β = 0.616, *p* < 0.001), supporting Hypothesis 3. The findings also revealed a significant and positive association between parenting style and academic motivation (β = 0.442, *p* < 0.001), thus supporting Hypothesis 5. Moreover, a significant and positive correlation was observed between academic self-efficacy and academic motivation (β = 0.642, *p* < 0.001), confirming Hypothesis 6. Lastly, academic motivation was found to significantly and positively predict academic resilience (β = 0.563, *p* < 0.001), verifying Hypothesis 7.

**Table 4:** Direct estimations.

H	Relationships			Standardized	*P* value
				Estimates	
H1	Parenting Style	→	Academic Resilience	0.396**	< 0.001
H2	Self-efficacy	↔	Academic Resilience	0.531**	< 0.001
H3	Parenting Style	→	Self-efficacy	0.616**	< 0.001
H5	Parenting Style	→	Academic Motivation	0.442**	< 0.001
H6	Self-efficacy	→	Academic Motivation	0.642**	< 0.001
H7	Academic Motivation	→	Academic Resilience	0.563**	< 0.001

Significant *p < 0.05, **p < 0.001.

### Mediation analysis

In order to determine the mediation influence of self-efficacy and academic motivation. The present study employed the Bootstrap approach to examine the mediating effect, utilising the AMOS programme. The study conducted 5000 iterations of sampling using the Bias-corrected technique and Percentile method tests. The results indicated that both the direct and indirect effects fell within the 95% confidence interval. The top and lower bounds of the analysis did not encompass zero values, with a significance level of *p* < 0.01. This suggests that the model can be classified as a partial mediation model, as seen by the findings presented in Table 5.

**Table 5:** Indirect analysis.

Path	Effect	S.E.	*P* value	Bias-corrected 95% CI		Percentile 95% CI	
				Lower	Upper	Lower	Upper
PS → SE → AR	0.210	0.049	0.001	0.198	0.297	0.203	0.366
PS → AM → AR	0.245	0.037	0.001	0.118	0.267	0.117	0.281
PS → SE → AM → AR	0.153	0.076	0.014	0.092	0.175	0.083	0.167

*PS* parenting style, *SE* self-efficacy, *AM* academic motivation, *AR* academic resilience.

According to the findings presented in Table 5, the 95% confidence interval parenting style is linked to academic resilience, as measured by self-efficacy, which ranges from 0.209 to 0.389. These values, which do not include 0, suggest that there is a significant mediating influence. Furthermore, the *p* value of less than 0.05 supports the conclusion that hypothesis H4 is valid. The 95% confidence interval for the influence of parenting style on academic resilience, in terms of academic motivation, ranged from 0.117 to 0.281. This interval does not include the value of 0, and the p-value was less than 0.05, showing that the mediating impact was statistically significant. Therefore, hypothesis H8, which posits the existence of a mediating effect, is supported. The study found that there were significant indirect effects and chain mediation in the relationship between parenting style and academic resilience. The upper and lower limits of self-efficacy and academic motivation were determined to be 0.083 and 0.175 at a 95% confidence interval. These values were found to be statistically significant, with *p* values less than 0.05. Therefore, hypothesis H9 was supported.

## Discussion

This study looked to examine the relationship between parenting style and academic resilience among college students. This study looked at the direct association between parenting style, motivation, and academic resilience in university students for the first time. In addition, the study sought to investigate the mediating functions of self-efficacy and academic motivation in the relationship between parenting style and academic resilience. Four latent variables, nine research hypotheses, and social cognitive theory were considered in this study. The hypothesized model was tested using structural equation modelling to explain the relationships, mediation, and causality between variables. According to the results of the study, social cognitive theory is capable of being utilized to explain the behaviours of academic resilience. The results are summarized as follows:

Regarding the initial hypothesis, the findings revealed a direct and positive correlation between parenting style and students' academic resilience. This finding is consistent with the study results presented in Mahdavi Mazdeh, Hejazi^71^ and Çakmak Tolan and Bolluk Uğur^72^, which suggest that parenting style plays a constructive role in developing adolescents' resilience. One possible reason is that parenting support and boundaries are balanced, which may be one factor. They nurture and support their children emotionally while also having high expectations for them. This parenting approach encourages independence and self-discipline in adolescents, which may help them overcome obstacles and setbacks in their education. Emotionally supportive parents provide an environment in which their children feel secure enough to express their feelings and seek help when necessary^73^. This emotional support assists students in dealing with the stresses, anxieties, and disappointments that are prevalent in academic environments. Moreover, parenting styles are complicated and multifaceted. They may not be completely captured by the study's approach. Furthermore, parenting methods might shift over time or vary between circumstances or siblings. Changes in living circumstances, such as finances, marital status, health concerns, or relocation, can all have a big impact on ways of parenting. In uncertain times, parents may become more protective, whereas in stable times, they may allow more autonomy. The findings of this study support the importance of parenting style for students' academic resilience.

Concerning the first research hypothesis, the findings indicate a positive correlation between parenting style and self-efficacy. One potential rationale for this observation is that adolescents frequently acquire knowledge through the process of observing and emulating their parents. When parents exhibit confidence, problem-solving abilities, and perseverance when confronted with difficulties, it is more probable that adolescents will internalize these characteristics and cultivate a feeling of self-efficacy. This study found that when youngsters are consistently provided with positive reinforcement and constructive feedback, they are more inclined to develop a sense of self-efficacy. This finding supports several previous studies^74–76^. The third hypothesis is that self-efficacy is positively associated with academic resilience. The results of this study have substantiated the notion that self-efficacy plays a crucial role in determining the academic resilience of students. One possible explanation for this outcome is that individuals with elevated levels of self-efficacy tend to possess a more optimistic perspective regarding their capabilities and their ability to surmount obstacles. The adoption of a positive mindset can enhance an individual's resilience by facilitating the sustenance of motivation and hope in challenging circumstances. Furthermore, students who possess a strong belief in their capacity to attain their academic objectives demonstrate a greater likelihood of persevering in their endeavours, especially in the face of obstacles^77^. This attribute is considered a fundamental component of academic resilience. Furthermore, this study contributes to literature on social cognitive theory by demonstrating the mediating roles of self-efficacy in parenting style and students’ academic resilience (H4). Logically, the existing correlation between parenting style and self-efficacy, along with the connection between self-efficacy and academic resilience, suggests that self-efficacy serves as a plausible mediator in the association between parenting style and academic resilience. This logical matter is proved by statistical mediating analysis. The significance of self-efficacy in improving students' academic resilience was further proven by its emergence as a significant mediating factor in the study. However, social cognitive theory offers a simple framework for comprehending complicated human activities. This paradigm may not fully reflect the multidimensional character of notions such as self-efficacy and parenting style. Moreover, social cognitive theory originated predominantly in Western cultures. Applying it globally may fail to account for cultural differences in parenting approaches and self-efficacy development. Cultural norms and values can have a substantial impact on both constructs. The idea might not be applicable to all demographic groups. Age, financial background, and family structure can all influence self-efficacy and parenting approaches, and these aspects may not be properly accounted for in social cognition theory-based research.

The subsequent hypotheses, H5 and H6, aim to ascertain the relationship between parenting style, self-efficacy, and academic motivation. This investigation lends support to some earlier research^21,34,47,49^. One potential elucidation Individuals who possess a strong sense of self-efficacy tend to establish academic objectives that are both ambitious and attainable, hence fostering their motivation to actively pursue and accomplish said goals. Individuals are inclined to exert greater effort, demonstrate perseverance in the presence of obstacles, and perceive setbacks as occasions for personal growth. Students who possess a strong sense of self-efficacy have enhanced confidence in their academic capabilities, resulting in heightened levels of engagement and a propensity to confront intricate issues. A parenting style characterised by support, autonomy promotion, encouragement, and guidance has been found to cultivate a more favourable academic motivation in children. There is a positive correlation between parenting support and intrinsic motivation for academic success among students^78^. On the contrary, it is worth noting that parenting approaches characterized by a lack of support or excessive control may exert a detrimental influence on academic motivation. These various techniques of teaching can potentially result in emotions such as stress, apprehension, or a diminished sense of motivation towards the process of acquiring knowledge. This study, associated with H4, aligns with Bandura's social cognitive theory, which posits that an individual's cognitive processes are influenced by environmental circumstances and behaviour, ultimately linking with academic motivation. Jehanghir, Ishaq^79^ posited that social cognitive theory offers a comprehensive framework for comprehending academic motivation, with a specific focus on the cognitive parenting style perspective. Furthermore, the outcomes of this study corroborate the initial discoveries made by Deng, Zeng^57^, Lin, Longobardi^58^, and Abdolrezapour, Jahanbakhsh Ganjeh^8^, who provided evidence for the benefit of self-efficacy linked to academic motivation.

The following hypothesis, H7, is that academic motivation positively influences academic resilience. The result confirmed and supported the findings of Zhang^80^ and Skinner^81^, who conducted research in the past. Academic motivation is the propelling force that fuels effort, positive thinking, goal setting, resourcefulness, and adaptability, which is the fundamental explanation for this finding. All of these elements are essential for academic resilience. Motivated students are better equipped to confront and overcome academic obstacles, making motivation a significant contributor to their academic resilience as a whole.

The findings of the study showed that academic motivation is another significant partial mediating variable, which is consistent with the claim made by this study. This study confirmed that the significance of motivation is underlined in the relationship between academic resilience and parenting style. Students who are motivated are more likely to put in consistent effort to achieve the academic goals they have set for themselves. They are internally motivated to achieve success and are willing to put in long hours of labour even when confronted with obstacles. According to Skinner^81^, students who put in persistent effort find it easier to push through challenging situations. This is one factor that contributes to academic resilience. In a similar vein, this finding is in line with the findings of another piece of research, which found that the interactions between parents and adolescents can be beneficial in the creation of a calming and supportive family environment, which in turn enables students to strengthen their academic motivation and maintain their resilience throughout their academic journey^82^. These positive features of parenting style can lead to the development of strong academic motivation in a student. For example, if a student's parents are very supportive, provide encouragement, and emphasise the significance of education, then the student is more likely to be motivated to do well in school. This motivation, in turn, helps the student improve their capacity to endure despite academic challenges and disappointments, which eventually contributes to the student's academic resilience. In conclusion, the study suggested that the link between academic incentives, parenting style, and academic resilience once existed.

The results of the study also indicated that self-efficacy and academic motivation played a mediating role in a sequential manner, which is a very noteworthy outcome. This finding suggests that the influence of parenting style on academic resilience is mediated by self-efficacy and academic motivation in a sequential manner. Within the context being discussed, three notable mediating roles have been identified. The mediating role of self-efficacy is the largest, which is consistent with the notion that self-efficacy is the most essential factor in influencing behaviour change. In addition, this result is similar to that of Findyartini, Greviana^83^, who concluded that self-efficacy is the most influential factor in the relationship between parenting style and academic resilience.

In addition, it was discovered that adolescents' self-efficacy contributed more to academic motivation than parenting style. This may suggest that academic motivation was primarily derived from adolescents' self-efficacy in the resilience process due to their perceived ability to overcome obstacles in academic resilience^84^. By analysing the complex relationships between parenting style, self-efficacy, academic motivation, and academic resilience based on social cognitive theory, the results of this study may contribute to the advancement of knowledge regarding resilience behaviours.

## Conclusions

This article adds to the body of research in two ways that are theoretical. On the one hand, this study's results show that parenting style has a positive correlation with academic resilience. This may help us learn more about how parenting style affects academic resilience. In particular, adolescents who can get care from their parents may change the ways they deal with stress and become more resilient while at college^71^. However, the study found that self-efficacy and academic motivation may help explain how parenting style is linked to academic resilience. This adds to what is known about academic resilience. The study suggests that self-efficacy and academic motivation can pass on the positive effects of parenting style to youngsters, making them more resilient in university. Students who have higher levels of self-efficacy and academic motivation will be in a better mental state when it comes to resilience behaviours^9^. Students' personal factors self-efficacy and academic motivation can be boosted by a resilient environment with a good parenting style. This, in turn, helps them be more resilient in university. In the real world, the study can help parents better understand how students can stay motivated in school by looking at parenting style and other environmental factors as well as personal factors like self-efficacy. Concerning parenting style, parents should get the training, talks, and symposiums they need to understand how important it is to have a good parenting style and get better at making friends with youngsters^85^. In terms of self-efficacy, adolescents should be given ways to build their confidence and take an active role in building their resilience. Also, educational programs should boost the confidence of students, so they can handle any problems that come up in resilient conditions.

## Limitations and future studies

Future researchers should take into account the potential limitations of this work.

- The study primarily employed a cross-sectional study design, which limits our ability to establish causal relationships between the variables. A sample taken at one moment in time, especially a framework with the mediator(s), may not be representative of the population at other points in time. If the sample does not adequately reflect the larger population, this can lead to biased conclusions. Subsequent investigations may prioritize longitudinal studies as a means to delve into the correlation between parenting style and academic resilience.
- Self-reported metrics will always be subjective. This tendency for people to present themselves in a more favorable light, whether conscious or unconscious, is known as social desirability bias. Furthermore, self-reported statistics rely on the person's ability to correctly remember and record feelings, behaviors, or experiences. This recall may not be perfect, which could imply that the information is incorrect. Use qualitative techniques like focus groups and conversations. Some of these can provide more information about what students have gone through and how they perceive things than a questionnaire.
- The suggested theoretical model was exclusively evaluated in relation to a sample that was picked from four colleges, thereby restricting the extent to which the findings might be generalized. In order to enhance the credibility and reliability of the model, it is imperative to conduct additional validation using varied samples obtained from a broader range of universities in future research endeavors.
- This study aims to investigate the relationship between parenting style and academic resilience, specifically focusing on the mediating roles of self-efficacy and academic motivation. Nevertheless, it is important to acknowledge that there are additional variables that have influence on academic resilience, including but not limited to academic stress, self-esteem, and peer relationships. In order to enhance the persuasiveness of findings and recommendations for practical application, future research endeavours should incorporate a broader range of variables.
- Different ages and genders perform differently on regulatory emotional self-efficacy, and future study can differentiate the association of academic resilience on regulatory emotional self-efficacy based on age and gender.

### Supplementary Information

Supplementary Information 1.

Supplementary Information 2.

## Data Availability

The data are not publicly available due to the University of Malaya Research Ethics Committee rules and regulations. The data that support the findings of this research are available upon reasonable request from the corresponding author and with permission of the University of Malaya Research Ethics Committee.

## Abbreviations

SEM: Structural equation modelling
AVE: Average variance extracted
VIF: Variation inflation factor
C.R: Critical ratio
PS: Parenting style
SE: Self-efficacy
AM: Academic motivation
AR: Academic resilience
