# Supplementary material for: Academic resilience, self-efficacy, and motivation: the role of parenting style
Source: Sci Rep. 2024 Mar 6;14:5571. doi: 10.1038/s41598-024-55530-7 (PMC10918079; doi:10.1038/s41598-024-55530-7)
Supplement: Supplementary file 1 — Supplementary Information 1. [file 41598_2024_55530_MOESM1_ESM.docx]

**Supplementary Figures:**


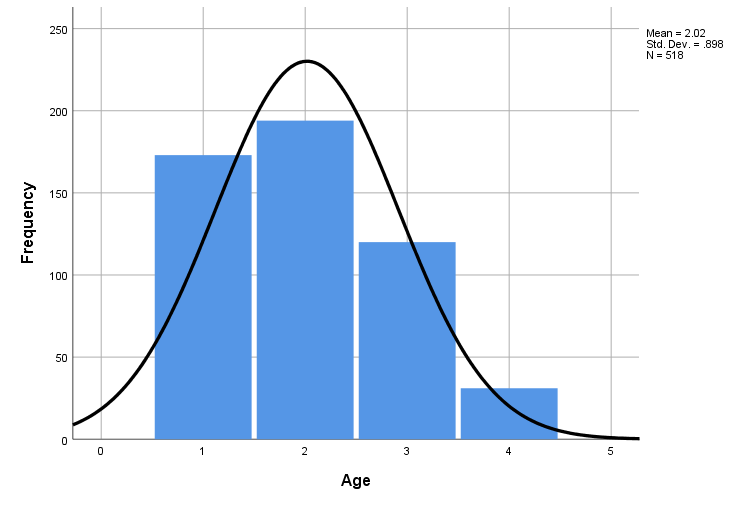


**Supplementary Figure 1:** Histogram of age distribution


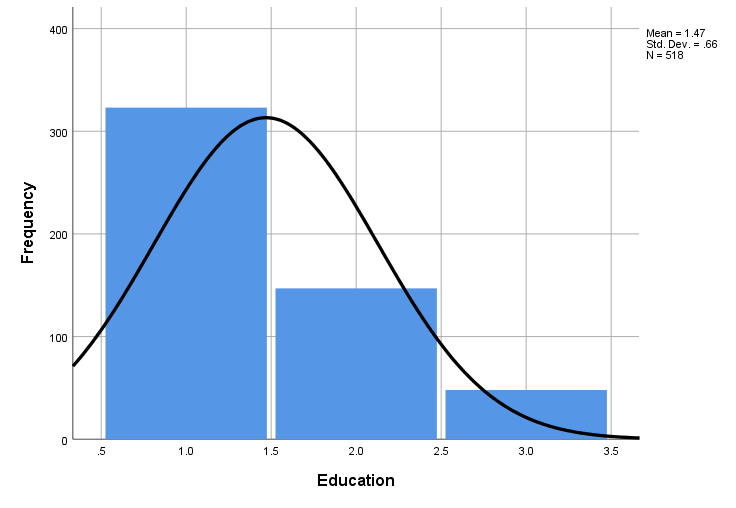


**Supplementary Figure 2:** Histogram of education distribution


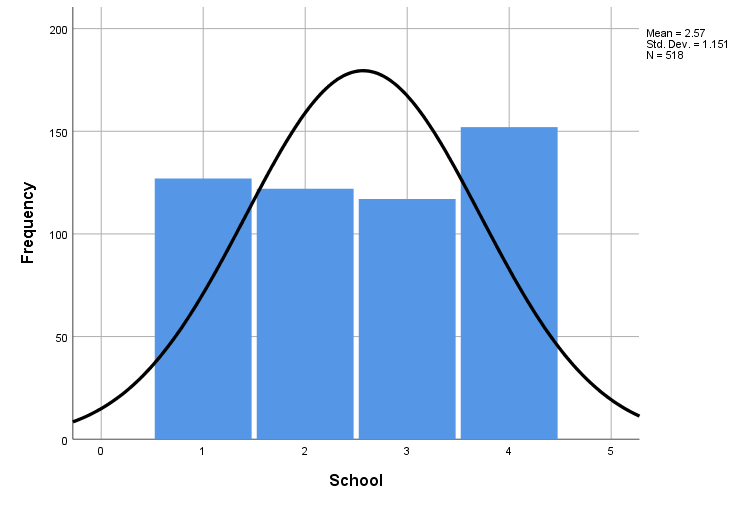


**Supplementary Figure 3:** Histogram of school distribution
