# Supplementary material for: Academic resilience, self-efficacy, and motivation: the role of parenting style
Source: Sci Rep. 2024 Mar 6;14:5571. doi: 10.1038/s41598-024-55530-7 (PMC10918079; doi:10.1038/s41598-024-55530-7)
Supplement: Supplementary file 2 — Supplementary Information 2. [file 41598_2024_55530_MOESM2_ESM.docx]

|  | **Academic Motivation items and factor loading** |  |  |
| --- | --- | --- | --- |
| **No** | **Item** | **Factor Loading** | |
|  | **Intrinsic Motivation Scale** |  |  |
|  | ***Challenge*** |  |  |
| 1 | I like hard work because it’s a challenge. | 0.638 |  |
| 2 | I like to learn as much as I can in school. | 0.786 |  |
| 3 | I like to go on to new work that’s at a more difficult level. | 0.617 |  |
| 4 | I like those school subjects that make me think pretty hard and figure things out. | 0.663 |  |
| 5 | I like difficult problems because I enjoy trying to figure them out. | 0.764 |  |
| 6 | I like difficult schoolwork because I find it more interesting. | 0.806 |  |
|  | ***Curiosity*** |  |  |
| 7 | I ask questions in class because I want to learn new things. | 0.729 |  |
| 8 | I do extra projects because I can learn about things that interest me. | 0.572 |  |
| 9 | I read things because I am interested in the subject. | 0.588 |  |
| 10 | I do my schoolwork to find out about a lot of things I’ve been wanting to know. | 0.754 |  |
| 11 | I work really hard because I really like to learn new things. | 0.65 |  |
| 12 | I work on problems to learn how to solve them. | 0.612 |  |
|  | ***Independent mastery*** |  |  |
| 13 | I like to try to figure out how to do school assignments on my own. | 0.804 |  |
| 14 | When I don’t understand something right away I like to try to figure it out by myself. | 0.791 |  |
| 15 | When I make a mistake I like to figure out the right answer by myself. | 0.697 |  |
| 16 | If I get stuck on a problem I keep trying to figure out the problem on my own. | 0.584 |  |
| 17 | I like to do my schoolwork without help | 0.733 |  |
|  | **Extrinsic Motivation Scale** |  |  |
|  | ***Easy work*** |  |  |
| 18 | I don’t like to figure out difficult problems. | 0.652 |  |
| 19 | I like to learn just what I have to in school | 0.781 |  |
| 20 | I don’t like difficult schoolwork because I have to work too hard. | 0.766 |  |
| 21 | I like easy work that I am sure I can do. | 0.645 |  |
| 22 | I like to stick to the assignments which are pretty easy to do. | 0.735 |  |
| 23 | I like school subjects where it’s pretty easy to just learn the answers. | 0.682 |  |
|  | ***Pleasing teacher*** |  |  |
| 24 | I read things because the teacher wants me to. | 0.663 |  |
| 25 | I do my schoolwork because teacher tells me to. | 0.659 |  |
| 26 | I work on problems because I’m supposed to. | 0.594 |  |
| 27 | I ask questions because I want the teacher to notice me. | 0.696 |  |
|  | ***Dependence on teacher*** |  |  |
| 28 | When I don’t understand something right away I want the teacher to tell me the answer | 0.762 |  |
| 29 | I like to have the teacher help me with my schoolwork. | 0.651 |  |
| 30 | When I make a mistake I like to ask the teacher how to get the right answer. | 0.708 |  |
| 31 | If I get stuck on a problem I ask the teacher for help. | 0.799 |  |
| 32 | I like the teacher to help me plan what to do next. | 0.659 |  |
| 33 | I like to ask the teacher how school assignments should be done. | 0.744 |  |

|  | **Academic Self-efficacy items and factor loading** |  |
| --- | --- | --- |
| **No** | **Item** | **Factor Loading** |
| 1 | I am convinced that I am able to successfully learn all relevant subject content even if it is difficult. | 0.697 |
| 2 | I know that I can maintain a positive attitude toward this course even when tensions arise. | 0.692 |
| 3 | When I try really hard, I am able to learn even the most difficult content. | 0.746 |
| 4 | I am convinced that, as time goes by, I will continue to become more and more capable of learning the content of this course. | 0.666 |
| 5 | Even if I get distracted in class, I am confident that I can continue to learn well. | 0.745 |
| 6 | I am confident in my ability to learn, even if I am having a bad day. | 0.784 |
| 7 | If I try hard enough, I can obtain the academic goals I desire. | 0.696 |
| 8 | I am convinced that I can develop creative ways to cope with the stress that may occur while taking this course. | 0.784 |
| 9 | I know that I can stay motivated to participate in the course. | 0.658 |
| 10 | I know that I can finish the assigned projects and earn the grade I want, even when others think I can’t. | 0.66 |

|  | **Parental Style items and factor loading** |  |
| --- | --- | --- |
| **No** | **Item** | **Factor Loading** |
| 1 | While I was growing up my mother felt that in a well-run home the children should have their way in the family as often as the parents do. | 0.762 |
| 2 | Even if his children didn’t agree with her‚ my mother felt that it was for our own good if we were forced to conform to what she thought was right. | 0.813 |
| 3 | Whenever my mother told me to do something as I was growing up‚ she expected me to do it immediately without asking any questions. | 0.788 |
| 4 | As I was growing up‚ once family policy had been established‚ my mother discussed the reasoning behind the policy with the children in the family. | 0.645 |
| 5 | My mother has always encouraged verbal give-and-take whenever I have felt that family rules and restrictions were unreasonable. | 0.783 |
| 6 | My mother has always felt that what children need is to be free to make up their own minds and to do what they want to do‚ even if this does not agree with what their parents might want. | 0.791 |
| 7 | As I was growing up my mother did not allow me to question any decision she had made. | 0.724 |
| 8 | As I was growing up my mother directed the activities and decisions of the children in the family through reasoning and discipline. | 0.646 |
| 9 | My mother has always felt that more force should be used by parents in order to get their children to behave the way they are supposed to. | 0.726 |
| 10 | As I was growing up my mother did not feel that I needed to obey rules and regulations of behavior simply because someone in authority had established them. | 0.774 |
| 11 | As I was growing up I knew what my mother expected of me in my family‚ but I also felt free to discuss those expectations with my mother when I felt that they were unreasonable | 0.793 |
| 12 | My mother felt that wise parents should teach their children early just who is boss in the family. | 0.759 |
| 13 | As I was growing up‚ my mother seldom gave me expectations and guidelines for my behavior. | 0.643 |
| 14 | Most of the time as I was growing up my mother did what the children in the family wanted when making family decisions. | 0.695 |
| 15 | As the children in my family were growing up‚ my mother consistently gave us direction and guidance in rational and objective ways. | 0.593 |
| 16 | As I was growing up my mother would get very upset if I tried to disagree with her. | 0.68 |
| 17 | My mother feels that most problems in society would be solved if parents would not restrict their children’s activities‚ decisions‚ and desires as they are growing up. | 0.732 |
| 18 | As I was growing up my mother let me know what behavior she expected of me‚ and if I didn’t meet those expectations‚ she punished me. | 0.652 |
| 19 | As I was growing up my mother allowed me to decide most things for myself without a lot of direction from her. | 0.745 |
| 20 | As I was growing up my mother took the children’s opinions into consideration when making family decisions‚ but she would not decide for something simply because the children wanted it. | 0.749 |
| 21 | My mother did not view herself as responsible for directing and guiding my behavior as I was growing up. | 0.784 |
| 22 | My mother had clear standards of behavior for the children in our home as I was growing up‚ but she was willing to adjust those standards to the needs of each of the individual children in the family. | 0.638 |
| 23 | My mother gave me direction for my behavior and activities as I was growing up and she expected me to follow her direction‚ but she was always willing to listen to my concerns and to discuss that direction with me. | 0.615 |
| 24 | As I was growing up my mother allowed me to form my own point of view on family matters and she generally allowed me to decide for myself what I was going to do. | 0.75 |
| 25 | My mother has always felt that most problems in society would be solved if we could get parents to strictly and forcibly deal with their children when they don’t do what the yare supposed to as they are growing up. | 0.757 |
| 26 | As I was growing up my mother often told me exactly what she wanted me to do and how she expected me to do it. | 0.639 |
| 27 | As I was growing up my mother gave me clear direction for my behaviors and activities‚ but she was also understanding when I disagreed with her. | 0.753 |
| 28 | As I was growing up my mother did not direct the behaviors‚ activities‚ and desires of the children in the family. | 0.656 |
| 29 | As I was growing up I knew what my mother expected of me in the family and she insisted that I conform to those expectations simply out of respect for her authority. | 0.738 |
| 30 | As I was growing up‚ if my mother made a decision in the family that hurt me‚ she was willing to discuss that decision with me and to admit it if she had made a mistake | 0.725 |

| **Academic Resilience items and factor loading** | | |
| --- | --- | --- |
| **No** | **Item** | **Factor Loading** |
| **Perseverance** | | |
| 1 | I would work harder | 0.773 |
| 2 | I would keep trying | 0.749 |
| 3 | I would use the feedback to improve my work | 0.783 |
| 4 | I would just give up | 0.683 |
| 5 | I would try to think of new solutions | 0.719 |
| 6 | I would change my career plans | 0.774 |
| 7 | I would use the situation to motivate myself | 0.606 |
| 8 | I would not change my long-term goals and ambitions | 0.546 |
| 9 | I would see the situation as a challenge | 0.619 |
| 10 | I would look forward to showing that I can improve my grades | 0.591 |
| 11 | I would see the situation as temporary | 0.736 |
| 12 | I would do my best to stop thinking negative thoughts | 0.765 |
| 13 | I would blame the tutor | 0.578 |
| 14 | I would not accept the tutors’ feedback | 0.739 |
| **Reflecting and adaptive help-seeking** | | |
| 15 | I would try to think more about my strengths and weaknesses to help me work better | 0.582 |
| 16 | I would give myself encouragement | 0.724 |
| 17 | I would seek encouragement from my family and friends | 0.625 |
| 18 | I would try different ways to study | 0.649 |
| 19 | I would set my own goals for achievement | 0.827 |
| 20 | I would seek help from my tutors | 0.622 |
| 21 | I would start to monitor and evaluate my achievements and effort | 0.674 |
| 22 | I would start to self-impose rewards and punishments depending on my performance | 0.776 |
| 23 | I would use my past successes to help motivate myself | 0.608 |
| **Negative affect and emotional response** | | |
| 24 | I would feel like everything was ruined and was going wrong | 0.621 |
| 25 | I would begin to think my chances of success at university were poor | 0.711 |
| 26 | I would probably get depressed | 0.556 |
| 27 | I would be very disappointed | 0.582 |
| 28 | I would begin to think my chances of getting the job I want were poor | 0.707 |
| 29 | I would probably get annoyed | 0.766 |
| 30 | I would stop myself from panicking | 0.619 |
